# Mr. Jackson's Case of Difficult Parturition

**Published:** 1826-07

**Authors:** 


### CASE OF DIFFICULT PARTURITION.*

Numerous are the difficulties which occasionally attend the parturient state, especially in this advanced æra of civilization. The case which Mr. Jackson has detailed was a very puzzling one, and the true nature of the phenomena were (fortunately) not unravelled by the scalpel. A young married woman, had been six hours in labour with her second child, when Mr. J. saw her. He found the concavity of the sacrum completely filled by a soft tumour, which was situated behind the rectum, and pushed both that and the vagina against the arch of the pubes. What was to be done? The tumour could not be punctured, except through the posterior parietes of the rectum, which Mr. J. was unwilling to injure. By great exertion a foot was brought down along the side of the tumour, and ultimately the body—lastly, but with extreme difficulty, the head. The day but one after delivery, the bowels

---

* W. Jackson, Esq. of Sheffield. Repos. March, 1826.

were obstructed, and the urine could only be passed with great difficulty. On examining the rectum, Mr. J. ascertained a very perceptible fulness and fluctuation between the anus and os coccygis. A lancet was, therefore, pushed in about an inch from the latter: this was followed by about six pints of a limpid straw-coloured fluid, and succeeded by a most agonising pain in the head, which was relieved by the recumbent posture. It was necessary to repeat this operation many times; and, at one period, the woman's life appeared in great danger, as the slightest pressure caused severe suffering along the dorsal vertebræ. The fluid discharged about this time also, was of a deep brown colour, and tinged with blood. She complained of a numb sensation in the lower extremities, with heat, thirst, and quick pulse. Leeches were applied along the spine, and other antiphlogistic measures were pursued. Notwithstanding these formidable phenomena, a healthy condition of the parts was ultimately re-established, and she has since borne a child without difficulty.

It is hard to say what was the origin or precise nature of this complaint. Dr. Denman appears to allude to something of the kind, under the term "dropsy of the perineum;" but this is a vague expression. We are inclined to agree with Mr. Jackson in supposing the fluid to have come from a diseased ovarium.

---

